# Simultaneous vs. sequential treatment for smoking and weight management in tobacco quitlines: 6 and 12 month outcomes from a randomized trial

**DOI:** 10.1186/s12889-018-5574-7

**Published:** 2018-05-31

**Authors:** Terry Bush, Jennifer Lovejoy, Harold Javitz, Alula Jimenez Torres, Ken Wassum, Marcia M. Tan, Bonnie Spring

**Affiliations:** 1 Alere Wellbeing (a solely owned subsidiary of Optum), 999 Third Avenue Suite 2000, Seattle, WA 98104-1139 USA; 2 Arivale, Inc and Institute for Systems Biology, Seattle, WA USA; 3 SRI International, Menlo Park, CA USA; 4 Feinberg School of Medicine, Northwestern University, Chicago, IL USA; 5 Center for Behavior and Health, Institute for Public Health and Medicine, Feinberg School of Medicine, Northwestern University, Chicago, IL USA

**Keywords:** Smoking, Weight management, Quitlines

## Abstract

**Background:**

Smoking cessation often results in weight gain which discourages many smokers from quitting and can increase health risks. Treatments to reduce cessation-related weight gain have been tested in highly controlled trials of in-person treatment, but have never been tested in a real-world setting, which has inhibited dissemination.

**Methods:**

The Best Quit Study (BQS) is a replication and “real world” translation using telephone delivery of a prior in-person efficacy trial. Design: randomized control trial in a quitline setting. Eligible smokers (*n* = 2540) were randomized to the standard 5-call quitline intervention or quitline plus simultaneous or sequential weight management. Regression analyses tested effectiveness of treatments on self-reported smoking abstinence and weight change at 6 and 12 months.

**Results:**

Study enrollees were from 10 commercial employer groups and three state quitlines. Participants were between ages 18–72, 65.8% female, 68.2% white; 23.0% Medicaid-insured, and 76.3% overweight/obese. The follow-up response rate was lower in the simultaneous group than the control group at 6 months (*p* = 0.01). While a completers analysis of 30-day point prevalence abstinence detected no differences among groups at 6 or 12 months, multiply imputed abstinence showed quit rate differences at 6 months for:simultaneous (40.3%) vs. sequential (48.3%), *p* = 0.034 and simultaneous vs. control (44.9%), *p* = 0.043. At 12 months, multiply imputed abstinence, was significantly lower for the simultaneous group (40.7%) vs. control (46.0%), *p* < 0.05 and vs. sequential (46.3%), *p* < 0.05. Weight gain at 6 and 12 months was minimal and not different among treatment groups. The sequential group completed fewer total calls (3.75) vs. control (4.16) and vs. simultaneous group (3.83), *p* = 0.01, and fewer weight calls (0.94) than simultaneous (2.33), *p* < 0.0001. The number of calls completed predicted 30-day abstinence, *p* < 0.001, but not weight outcomes.

**Discussion:**

This study offers a model for evaluating population-level public health interventions conducted in partnership with tobacco quitlines.

**Conclusions:**

Simultaneous (vs. sequential) delivery of phone/web weight management with cessation treatment in the quitline setting may adversely affect quit rate. Neither a simultaneous nor sequential approach to addressing weight produced any benefit on suppressing weight gain. This study highlights the need and the challenges of testing intensive interventions in real-world settings.

**Trial registration:**

ClinicalTrials.gov Identifier: NCT01867983. Registered: May 30, 2013.

## Background

Cigarette smoking and obesity are the leading causes of preventable morbidity and mortality in the U.S [1, 2]. Even though quitting smoking leads to long term improvements in health and reduction in cancer and heart disease risk, cessation can lead to weight gain and obesity-related co-morbidities [3, 4]. Over an 8-year period, the average smoker who quits gains 8.8 (sd 6.4) kg of weight attributable to abstaining from tobacco, with highest weight gains observed among those with higher body mass index (BMI; 10.2–19.4 kg) [5]. Up to 10% of ex-smokers gain in excess of 10 kg [6–8].

Weight gain or the fear of weight gain can impede smoking cessation efforts. Therefore, several studies have been conducted to determine the impact on both tobacco abstinence and suppression of excess weight gain of adding weight management to tobacco cessation counseling [9–11]. In one study, Spring et al. evaluated whether adding a weight management intervention to a cessation program improved weight outcomes without harming the quit rate [11]. That trial showed that a sequential approach (weight management after smoking cessation) was more effective at suppressing weight gain than either simultaneous delivery of tobacco cessation and weight counseling or tobacco cessation counseling alone. Adding weight management did not reduce smoking abstinence. The Spring et al. study enrolled women only and offered an in-person, group-based intervention with provision of meal replacements. Provision of in-person treatment and meal replacement limits the treatment’s scalability and may explain its lack of widespread dissemination.

The effectiveness of delivering weight management treatment sequentially or simultaneously with tobacco cessation coaching needed to be tested at a population level. Quitlines provide a natural population-based laboratory to test approaches to help people quit smoking and control their weight. Moreover, two thirds of smokers who call quitlines are overweight or obese, and two thirds are concerned that quitting smoking will cause them to gain weight [12]. Understanding how to optimize both smoking cessation and weight gain reduction in the quitline setting is an important public health priority. Thus, the current study tests the impact of adding an evidence-based weight management intervention simultaneously with or sequentially after cessation treatment delivered via telephone quitlines. The study--called the Best Quit Study (BQS)--was modeled on Spring et al.’s successful efficacy trial and is the first attempt to replicate those findings using widely available phone and web-based behavioral programs. The primary objectives of this randomized controlled trial were to examine, in the context of a quitline setting, whether adding weight management calls to smoking cessation counseling reduces weight gain without harming cessation outcomes, and whether outcomes differ depending on whether weight counseling calls are delivered simultaneously with tobacco cessation treatment or afterward (sequentially).

## Methods

The BQS methods, study measures and interventions are described comprehensively in the published protocol paper [13] and reviewed briefly below. Research activities and human subjects’ protocols were approved by the Western Institutional Review Board and overseen by a Data Safety and Monitoring Board (DSMB). The study began in 2013 and ended in 2017.

### Setting

This study was implemented by Alere Wellbeing which provides tobacco quitline services to over 350,000 tobacco users per year in 26 states and over two hundred employer groups and health plans. In 2015 more than 400,000 people nationwide used a state quitline [14]. Alere Wellbeing also provides a phone/web based weight management program (Weight Talk®).

### Recruitment, eligibility and screening

Study participants were recruited from three state quitlines (Indiana, Maryland and North Carolina) and 10 commercial (employer-provided) quitlines and were screened for eligibility during registration with the quitline from August 2013 to December 2014. Participants were eligible if they were 18 years of age or older, had a body mass index (BMI) > = 18.5, smoked at least 10 cigarettes per day (cpd), wanted to quit smoking within 30 days and could speak and read English. We excluded those who smoked fewer than 10 cigarettes per day to match the eligibility criteria from the prior efficacy trial. Exclusion criteria were: pregnant, current substance abuse or psychosis, current diabetes, history of an eating disorder, recent or planned surgery for obesity, lack of internet access, unavailable for any two week period of time over the next six months or did not want to receive ten coaching calls.

### Consent, randomization and assessment

Candidates who met eligibility requirements and expressed interest in a research study about smoking and weight were transferred to a quit coach who described the study and obtained verbal informed consent to participate, while remaining blind to treatment assignment. Requiring written consent would have placed additional burden on participants, delayed treatment initiation and reduced the generalizability of study findings. We received approval from the Western Institutional Review Board to collect verbal consent. A random number generator allocated individuals to one of three treatment groups (cessation alone, simultaneous cessation and weight or sequential cessation followed by weight). A coach informed participants of what to expect during treatment and proceeded to deliver the content for the first session. Coaching calls were recorded and monitored throughout the study. Process and outcome data were collected at 6 and 12 months via web survey, by phone or by mail (see data collection below).

### Participants

**Fig. 1:**
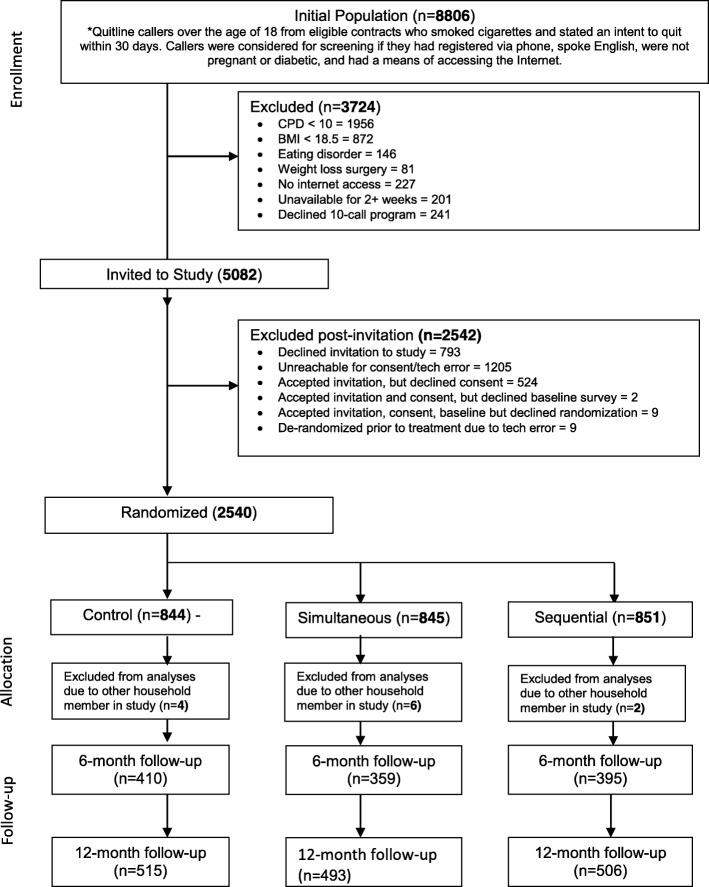
Best Quit Study CONSORT Diagram

Figure 1 shows the CONSORT diagram for this study. Of the 8806 smokers screened, 5082 were eligible and invited to the study; 3084 were interested and listened to the informed consent; 2560 consented and 2540 were randomized. Individuals (*n* = 3724) were excluded for the following reasons: smoked fewer than 10 cpd, low BMI defined as weight in kilograms divided by the square of the height in meters), current eating disorder, prior or planned weight loss surgery, no access to the internet, unavailable for two or more weeks or did not want to receive 10 coaching calls. Twelve individuals were later removed from the study when it was discovered that they did not satisfy the original exclusion criteria, leaving 2528 participants for analysis.

### Intervention and control arm procedures

**Fig. 2:**
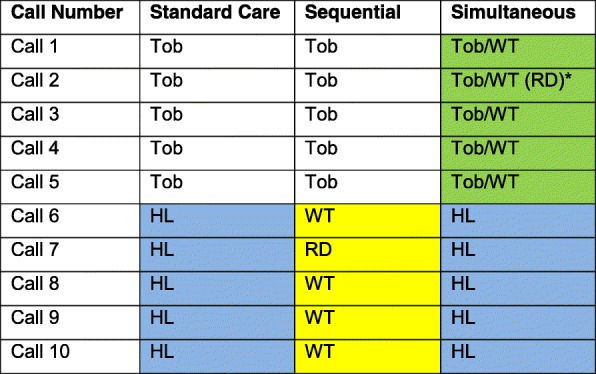
Typical five-call quitline schedule (Tob) with the added weight (WT) or healthy living (HL) calls^1^. Registered Dietician (RD) delivered the 2nd weight call^2^. 1. Individuals can call into the quitline for additional help at any time. 2. In the simultaneous group, a coach delivered the tobacco content and then transferred the call to an RD for the weight content

When a smoker called the quitline and enrolled in the standard 5 call counseling program, a registration agent collected demographic and smoking data and assessed the participant’s interest in the study. Those who wanted to hear more about the study were transferred to a coach who described the study and collected additional baseline data. Those who met study criteria and provided verbal informed consent to participate were randomized to treatment group and received their first counseling session. Smokers in all study arms received 10 coaching calls plus additional calls if requested. Coaches made several attempts per day over five different days to reach study participants to complete each of their 10 planned counseling sessions. Treatment was participant-focused, so the timing of each call varied by participant needs. The first “inbound” call was initiated by the smoker; “proactive” calls 2–10 were initiated by the coach. All participants could phone in to the quitline for additional support at any time. Those unscheduled calls were classified as ‘ad hoc’ calls. As shown in Fig. 2, the cessation only treatment group (control) received five standard quitline cessation calls followed by five healthy living program calls. The simultaneous group received five calls that combined cessation and weight management content followed by five healthy living program calls. The sequential group received five standard quitline cessation calls followed by five weight management calls. The healthy living calls acted as a contact control to equalize the number of contacts with a coach across all three groups. For the simultaneous group, since the second call in the weight program required a Registered Dietitian (RD), a quitline coach completed the standard tobacco content and then transferred the participant to an RD. This transfer was not needed for the sequential weight treatment. On the treatment calls for the simultaneous group, coaches were asked to integrate coaching elements applicable to both smoking cessation and weight management (e.g. offering strategies for choosing healthy foods, getting more physical activity and reducing stress). We anticipated that the counseling calls might be slightly longer (but not double) for simultaneous calls 1–5 than standard treatment because coaches were covering two content areas. Generally, the standard 5-call cessation program takes about two months to complete depending on participants schedule and preferences and timely response to the proactive coach-initiated calls. The additional five calls (Weight or Healthy Living control calls) took an additional 2–3 months to complete. The majority completed all 10 calls within 5 months.

### Standard tobacco treatment

Cessation treatment for all groups was the quitline intervention, which includes 5 counseling sessions with a coach plus unlimited call-ins to the quitline for help at any time, an interactive web intervention and a mailed Quit Kit containing a printed guide [15]. The guidelines-based counseling for smokers involved developing a quit plan, discussing and selecting medications, and devising quit strategies including creating a tobacco-free home; stress management, problem solving, behavioral skills training, and relapse prevention once abstinent [16, 17]. Counseling content is based on Social Cognitive Theory [17] and utilizes cognitive behavioral therapy, motivational interviewing techniques, problem solving and relapse prevention theory [18]. During this study, all of the participating quitlines also offered free nicotine replacement therapy (NRT) in the form of patch, gum and/or lozenge (0–8 weeks), depending on the contract and appropriateness based on the participant’s medical condition. Quitlines have been shown to be an effective and cost effective treatment for smokers [16].

### Weight management treatment (weight talk®)

Alere Wellbeing’s weight management program (Weight Talk®) was modified for the present study to include a reduced number of calls and to focus on weight gain prevention rather than weight loss. Thus, the weight management program involved 5 counseling calls offered by coaches and RDs, mailed materials and access to a web-based weight management program with on-line tracking forms, goal setting and educational components. Coaches encouraged participants to set diet, physical activity, and weight goals, regularly self-monitor their weight, dietary intake (e.g. calories), stress, and physical activity level. The second call of the weight management intervention (call 2 for simultaneous, call 8 for sequential) was delivered by an RD and covered topics on calorie reduction strategies and the rationale for why and how to reduce caloric intake. An RD was required for this call because of the higher level skills needed to help participants reduce their daily calorie consumption by approximately 300 cal, the amount that is likely to offset potential weight gain from a lower metabolism associated with smoking cessation. Our weight management program is grounded in Social Cognitive Theories and utilizes dietary and physical activity behavior change interventions proven to be efficacious in producing weight loss [19–22]. The dietary component was based on the DASH (Dietary Approaches to Stop Hypertension) eating plan [23]. Physical activity recommendations came from the American College of Sports Medicine and the Physical Activity Guidelines for Americans developed by the US Department of Health and Human Service [24]. The stress reduction component focuses on identifying and controlling stressful situations, finding and practicing coping skills and monitoring progress.

### Healthy living program calls

The 5 healthy living (control) calls addressed sun protection, flu prevention, pedestrian safety, disaster preparedness and home energy savings. Call content was meant to be neutral, in that coaches did not discuss tobacco or weight.

### Coaches and training

Interventions were delivered by experienced quitline coaches and RDs. Coaches who provided an intervention (tobacco, weight management, and/or healthy living) were trained to deliver that intervention. RDs were board certified and experienced. For all coaches, the training included reviewing the treatment manual, listening to tape recorded ‘mock’ calls and practicing the intervention content via role-plays. Coaches followed a structured pattern of counseling which covered specific intervention topics visible to coaches via their on-line coaching application. If a participant wanted to discuss tobacco cessation during calls devoted to weight management or healthy living, coaches were instructed to deliver the scheduled intervention content first and then deliver the tobacco content and record this portion of the call as an ‘adhoc tobacco’.

### Fidelity monitoring

Research staff listened to recorded calls from each coach and from each content area (Tobacco, Weight, Healthy Living) and provided feedback to ensure that coaches delivered the treatment as intended. Coders from the research team rated 222 calls throughout the trial to ensure compliance with the interventions and prevent drift. Staff created a study specific fidelity measure to evaluate four aspects of coaching quality using a 1–5 rating where 1 = Extremely poor, 3 = As expected, 5 = Excellent. The topics were: 1) Therapeutic Nonspecifics: Was the call facilitated well and with good rapport? 2) Tobacco and Non-Tobacco Content: Was the correct Tobacco or Healthy Living call content delivered? 3) Weight Content: Was the correct Weight Intervention call content delivered? 4) Contamination: Was content from an incorrect condition delivered? (e.g., Healthy Living Content on a Tobacco call, Weight content on a Tobacco only call). The criterion for adequate fidelity was set at a rating of 3 or higher. Average scores were: 3.42 for Therapeutic Nonspecifics, 3.34 for correct Tobacco and Healthy Living content, 3.41 for correct Weight intervention content, and 4.69 for absence of Contamination. Contamination occurred on only one occasion when a coach mistakenly began to discuss weight on a tobacco only call, but quickly realized his error and refocused the session on tobacco cessation. A booster training of all coaches occurred one time early in the study when fidelity fell below 80% of our compliance threshold of 3.

### Data collection

Participants’ demographic, smoking, and weight characteristics were collected during quitline registration (by a quitline agent). Baseline information was collected by a coach prior to randomization. Process and outcome data were collected at 6 and 12 months post-randomization by the survey team who were blinded to treatment arm. Participants could earn up to $110 for completing surveys ($30 for baseline; $35 for 6 month, $35 for 12 month and $10 for early completion of the 12 month web survey). Two weeks prior to the 6 and 12 month target dates, participants were sent an email with a link to the survey. Survey non-responders were sent reminder emails and those still nonresponsive were contacted by phone. Survey staff attempted telephone outreach for at least 11 days and left several voice messages asking the non-responding participant to call the quitline. Individuals who still did not respond were sent a mailed copy of the survey with a stamped return envelope. Those who failed to return the mailed survey within two weeks were sent a short form survey asking only four questions (satisfaction, tobacco status, cpd and current weight). The envelope stated that compensation was enclosed (a $2 bill was enclosed with the survey). Among the 1514 who completed the 12 month survey, 32.6, 29.6 and 30.0% of control, sequential and simultaneous groups respectively, completed the web survey; 62.0, 67.1 and 65.6% completed the survey by phone or mail and 5.4, 3.4 and 4.5% returned the short survey.

#### Response rate

Survey response rate at 6 months was significantly lower for simultaneous treatment (42.8%) as compared to cessation only (48.8%), *p* = 0.01, but did not differ from sequential treatment (46.5%), *p* = 0.12. Response rates at 12 months were similar across groups; 61.3% for controls, 59.6% for sequential and 58.8% for simultaneous groups, *p* = 0.55.

#### Utilization of treatment

In addition to self-reported data, treatment utilization was collected by Alere and included type and number of counseling calls completed (scheduled and participant initiated calls). Ad-hoc calls initiated either as a change of topic during a scheduled call or as separate participant initiated calls for assistance were tracked and labeled by content (Tobacco, Weight, and Healthy Living). We aimed for all participants to complete 10 calls: the five quitline counseling calls (tobacco) and the five group specific coaching calls (five weight management or five healthy living). Call 1 lasted 28, 29 and 39 min for control, sequential and simultaneous group, respectively. Calls 2–5 lasted about 13 min per call for control and sequential groups (tobacco content) and 16–22 min for the simultaneous group (tobacco/weight content). The RD call averaged 22 min for simultaneous group and 20 min for sequential group. Calls 6–10 lasted about 7 min per call for the control and simultaneous groups (the healthy living calls) and 15–20 min per call for the sequential weight calls. The number of adhoc calls also varied by group (six adhoc calls for controls, six for the sequential group and 10 for the simultaneous group). These adhoc calls averaged 84, 107 and 180 min, respectively for controls, sequential and simultaneous groups.For the simultaneous group, if content for call 2 could not be delivered in a single session (tobacco content followed by direct transfer to an RD) then a separate call initiated by the RD was needed and if completed, it was added to the total calls. Total ‘tobacco calls’ was the sum of all calls that included a tobacco intervention (i.e., both tobacco only calls and calls that combined a tobacco and weight intervention). Similarly, total ‘weight calls’ was the sum of all calls that included a weight intervention.

The simultaneous group completed fewer tobacco calls compared with controls and sequential (*p* < 0.001) but more weight calls than sequential (*p* < 0.0001) since these were integrated within the initial tobacco treatment calls. The sequential group completed fewer calls overall (Tobacco + Weight calls) than simultaneous Tobacco/Weight + Healthy Living calls) or controls (Tobacco + Healthy Living calls); *p* = 0.01.

### Study measures

Self-reported data obtained for this study were collected using standardized validated measures (where available) that have been used to assess tobacco use and cessation outcomes as well as demographic and weight related data in prior research studies [11, 25]. Screening data collected by a registration agent when a smoker called into the quitline included standard demographic and tobacco use questions (e.g. age, gender, chronic disease status, readiness to quit) plus study specific eligibility questions (e.g. cpd, height, weight, history of eating disorder, weight loss surgery, access to internet; reachable for next six months and willingness to take five additional counseling calls). Registration took about 10 min to complete. Baseline data collected by a coach took 3–4 min to gather responses to nine questions (race, ethnicity, education, marital status, depression/anxiety, if dieting, level of weight concerns and confidence in avoiding weight gain). The latter two questions were: “How concerned are you about gaining weight after quitting” and “How confident are you that you can avoid gaining weight while staying quit?” A score of 6 or higher using a 10 point scale (1 = Not at all and 10 = Extremely) was considered to reflect moderate weight concerns. Individuals were categorized as normal weight, overweight or obese using standard BMI cutoffs [26]. Depression and anxiety were measured with four questions using a 4-point scale where 0 = Not at all and 3 = Nearly every day to assess symptoms of anxiety, dysphoria, and anhedonia [27]. Content of the 6 and 12 month surveys included self-reported: duration of “no puff” abstinence, type and amount of tobacco used, cessation medications used, symptoms of depression or anxiety, satisfaction with the quitline, satisfaction with the study, current weight, and physical activity level (days/week and minutes/day of moderate physical activity in the past week).

#### Primary outcomes of the study

Primary outcomes were self-reported 30-day multiply imputed (MI) point prevalence abstinence (ppa) and change in weight at 6 months. Weight change was calculated as follow-up weight minus baseline weight and percent change (follow-up weight divided by baseline weight). *Secondary outcomes* were self-reported 30-day ppa where missing is coded as smoking and 30-day *observed* ppa (OBS) and change in weight among survey respondents at 6 and 12 months. Other outcomes were reduction in number of cigarettes smoked and satisfaction with treatments.

The original proposal was to conduct two sets of comparisons: 1) a test of the combined treatment groups against the control group, 2) a test of one treatment group against the other treatment group. Due to implementation issues (discussed below) we made a slight change and rather than combining the two treatment groups together in the regressions (i.e., have a single indicator variable for any weight management treatment) we allowed each weight management treatment group to have its own indicator variable in the regression and then tested for the combined statistical significance of the regression coefficients for the two indicators (using a Wald chi-square test with 2 df).

#### Extreme outliers of self-reported weight

In this study we relied upon a person’s accuracy in estimating their current weight at baseline, 6 and 12 months and correctly documenting this report in the online, mailed or phone surveys. It is possible that our methods allowed for participant and/or data collection error. We therefore looked for extreme outliers defined as a clinically unusual change in weight of 22.7 kg (50 pounds) or greater over a 6 month period or 45.3 kg (100 pounds) or greater over 12 months and excluded these data from the analyses of weight. Thus, we omitted 28 participants from the 6 month and 10 from the 12 month analyses. There were no differences among treatment groups on number of outliers excluded. We then analyzed the data with and without these extreme outliers and again found no significant differences by treatment condition on change in weight. Excluding outliers changed the average percent weight gain by less than .02% across all groups, and by no more than .43% for any individual group.

### Statistical analysis

*Hypotheses* were based on the previous efficacy findings in which the sequential group reported significantly less weight gain than cessation only or the simultaneous group [11]. Thus, we hypothesized that: 1) the sequential group would have lower cessation related weight gain than the control or simultaneous groups and 2) cessation rates would not be statistically significantly different across groups. We pre-defined treatment success as a significant improvement relative to the control group in either 30-day abstinence or weight suppression at 6 or 12 months, without harming the other outcome.

The primary analysis approach for testing the effectiveness of weight management treatment groups on cessation was logistic regression with separate indicators for each treatment group followed by Wald’s test that both treatment group regression coefficients were zero. Analyses of cessation were conducted separately at 6 and 12 months in three ways: 1) on the set of responders to follow-up (i.e., all available respondents for whom we know the cessation outcome; (OBS)), 2) assuming that non-responders (lost to follow-up) were current smokers (imputing missing = smoking), and 3) using multiply imputed values for missing cessation status (MI). Assuming missing data to be indicative of relapse is a standard method used in population-based smoking cessation studies. It is based on the assumption that relapsers are less likely to respond to follow-up surveys due to their disengagement from the quitting process. We recognize that it is overly deterministic. Because there is little evidence concerning the accuracy of this method, we have included it as one of three approaches to calculating the abstinence rate. We used a two-sided hypothesis test to identify any statistically significant effect on cessation or weight outcomes. We addressed missing weight values using multiple imputation as well as sensitivity analyses with different assumed amounts of weight gain for non-responders. Regressions were conducted for all participants and separately for relapsers and quitters. Analyses for both smoking and weight outcomes included indicators for each treatment group. For the outcome of smoking, the covariates were baseline BMI, gender, age, cpd, time to first cigarette, confidence in quitting, depressive symptoms and state vs. commercial quitline. These covariates have been shown to impact smoking cessation [28]. For the outcome of weight gain, the covariates were baseline BMI, gender, age, cpd, depressive symptoms, weight concerns, confidence in avoiding weight gain, and state vs. commercial quitline.

Preliminary analyses included summary statistics (mean, standard deviation, skewness, kurtosis, range), cross tabulations of baseline variables and outcomes versus group, correlations among variables, and tabulations of outcome variables versus time. Standard regression diagnostics included examination for heteroscedasticity, outliers, observations with large leverage, observations with large influence, and normality of residuals (where appropriate). Plots (such as residuals versus covariates and residuals versus fitted values) were examined for the model fit. For all outcomes we conducted a 2 degree of freedom test for any difference between groups, and also tested for any differences between each pair of groups. We also explored the interaction of treatment group and select covariates shown above on abstinence and percent change in weight. We conducted descriptive analyses of other data including treatment utilization, defined as number of counseling sessions completed per type of content delivered (Tobacco, Weight or Healthy Living).

## Results

### Participants

There were no significant demographic differences among the groups. Randomized participants were 66% female, 68% white, 23% Medicaid-insured, 76% overweight/obese and between the ages of 18 and 86 and mean age = 43.2 ± 12.2 [29].

### Smoking abstinence

As shown in Table 1, the primary 6 month smoking outcome of multiply imputed 30-day ppa was not significant overall (*p* = 0.08). Bivariate analyses showed that the simultaneous group had significantly lower multiply imputed abstinence than control (*p* = 0.04) and sequential treatment (*p* = 0.03). Similarly, using missing = smoking, the simultaneous group again had significantly lower abstinence than the other two groups (*p* = 0.02). For the 12 month follow-up, the analysis using multiply imputed data continued to show reduced abstinence for the simultaneous group as compared to cessation only controls (p = 0.04). Not shown are the non-significant treatment effects in the observed abstinent rates. At 6 months, abstinence rates were 52.7; 53.7, and 47.2 for control, sequential and simultaneous treatments, *p* = 0.18. At 12 months, abstinence rates were 47.8, 46.5, and 42.6 for control, sequential and simultaneous treatments, *p* = 0.24. Analyses of the interaction of treatment group with various covariates on 30-day abstinence at 6 months showed that the only statistically significant interaction was for the sequential group with baseline BMI (*p* = 0.05) indicating that those with higher BMI were less likely to quit. At 12 months, the only statistically significant interaction effects were for: 1) the sequential group with baseline BMI, (*p* = 0.05) on 30-day observed abstinence; and 2) the simultaneous group with time to first cigarette after waking (*p* = 0.005). Thus, for the sequential group, those with higher BMI had lower quit rates and for the simultaneous group, those who were more addicted (earlier time to first cigarette) were less likely to quit smoking. However, these interactions were not significant based on multiple imputation analyses.

**Table 1:** 30-day point prevalent abstinence at 6 months and 12 months by treatment group

	Control *N* = 840	Sequential *N* = 849	Simultaneous *N* = 839	Weight management treatment vs control: Wald’s X2; *p*-value
6-month outcomes				
% abstinent (Multiply Imputed)	44.9	48.3	40.3	2.94; *p* = 0.08^a^
% abstinent (Missing = Smoking)	24.4	23.8	19.2	7.84; *p* = 0.02^b^
12-month outcomes				
% abstinent (Multiply Imputed)	46.0	46.3	40.7	1.83, *p* = 0.16^c^
% abstinent (Missing = Smoking)	28.2	26.9	24.1	3.89, *p* = 0.14

^a^There was a statistically significant difference between the simultaneous and control groups (*p* = 0.036) and between simultaneous and sequential (*p* = 0.032).

^b^There was a statistically significant difference between the simultaneous and sequential groups (*p* = 0.024) and between the simultaneous and control groups (*p* = 0.01).

^c^Simultaneous group was significantly different from controls (*p* = 0.039)

### Smoking reduction among continuing smokers

There were no significant treatment differences on change in the amount smoked from baseline to 6 or 12 months. The mean (sd) reduction in cpd among continuing smokers was 8.9 (9.5) at 6 months and 7.9 (9.5) at 12 months.

### Weight change

Table 2 shows the mean (sd) change in weight in kgs from baseline to 6 or 12 months for each treatment group. Results indicate no significant difference between groups on change in weight, even among those who had quit smoking. Findings were similar for absolute weight change and percent weight change from baseline to follow-up, and analyses controlling for covariates confirmed these findings. The wide variability in amount of weight change is depicted by the large standard deviations and standard errors shown in the tables. Change in weight from baseline to follow-up (excluding weight outliers) ranged from a 20.4 kg loss to a 20.4 kg gain at 6 months and from a 40.8 kg loss to a 44.9 kg gain at 12 months. At 6 months, 14% lost > 4.5 kgs and 23% gained > 4.5 kgs. At 12 months 19% lost > 4.5 kgs and 26% gained > 4.5 kgs.

**Table 2:** Calculated change in weight in kgs at 6 and 12 months (excluding outliers)^a^

	Control *N* = 369	Sequential *N* = 338	Simultaneous *N* = 312	Statistics
6-month outcomes				
Mean (sd), se (Multiply Imputed) *n* = 2374	−0.22 (6.9), 0.39	0.75 (7.8), 0.41	0.004 (7.5), 0.38	F(2, 106) = 1.52; *p* = 0.22
Mean (sd), se (completers) *n* = 1019	0.11 (5.4), 0.28	0.60 (5.99), 0.23	0.19 (5.5), 0.31	F(2, 1016 = 0.75; *p* = 0.47
Mean (sd), se (among abstinent *n* = 521	0.35 (5.8), 0.42	0.56 (6.2), 0.46	0.45 (6.0), 0.49	F(2, 518) = 0.06, *p* = 0.94
12-month outcomes				
Multiple imputation: Mean (sd), se (all *n* = 2520)	−0.22 (9.2), 0.40	0.41 (9.4), 0.40	0.40 (7.9), 0.36	F2(2, 264) = 0.87;*p* = 0.42
Mean (sd), se (completers) *n* = 1399	−0.18 (8.4), 0.38	0.34 (8.2), 0.38	0.27 (7.4), 0.34	F(2, 1396) = 0.58, *p* = 0.56.
Mean (sd), se (among abstinent *n* = 628	−0.38 (9.7), 0.65	0.44 (9.7), 0.63	0.40 (8.2), 0.59	F(2, 625) = 0.57; *p* = 0.57

^a^ *sd = standard deviation; se = standard error*

*Note: at 6 months 13.9% lost > 4.5 kg and 23.3% gained > 4.5 kg*

Also, for multiple imputation the df is completely different than the number of observations *Note: at 12 months 19% lost > 4.5 kg and 26% gained > 4.5 kg*

### Satisfaction

Ratings of satisfaction with the quitline and satisfaction with study participation were high (mean > 5.1 on a 6 point Likert scale). Although analyses using all available data showed no group differences at 6 or 12 months, multiply imputed data including those with missing data at 12 months showed greater satisfaction with the quitline for the sequential group as compared to the simultaneous group (mean 5.3 vs. 5.1; *p* = 0.003) and compared with the cessation only controls (mean = 5.3 vs. 5.1; *p* = 0.02). Similarly, analyses of multiply imputed data at 12 months, showed the sequential group to be more satisfied with their study participation than the simultaneous group (mean = 5.3 vs. 5.1; *p* = 0.01).

### Treatment receipt

Total number of calls completed varied by group, 4.2 (control group), 3.8 (sequential group), 3.8 (simultaneous group). A greater number of calls completed was a significant predictor of better cessation rates at 6 months (*p* < 0.001). For every additional call completed, participants were 56–81% more likely to be quit at 6 months (adjusting for total number of minutes and web logins). Total calls was not a statistically significant predictor of percent weight change at 6 months, but was a significant predictor of percent weight change at 12 months [0.173% [0.030–0.315%), *p* = .02] indicating an increase in weight per call completed (possibly related to the increased chance of cessation per additional call). Total calls involving weight was not a statistically significant predictor of percent weight change at 6 or 12 months (Table 3).

**Table 3:** Participation rates: Mean (sd), range in number of calls completed, by Group

Mean(sd) se range	Control *N* = 840	Sequential, *n* = 849	Simultaneous *n* = 839	*P* values
Total Tobacco	2.83 (1.62) 0.06 0–8	2.81 (1.6) 0.06 0–10	2.56 (1.65) 0.06 0–14	F(2, 2525) = 7.23; *p* = 0.0007^a^
Total Weight	0	0.94 (1.54) 0.05 0–7	2.33 (1.52) 0.05 0–6	F (1,1686) = 349.0; *p* < 0.0001^b^
Total healthy living	1.33 (1.82) 0.06 0–5	0	1.05 (1.67) 0.06 0–5	F (1,1679) = 11.02; *p* = 0.0009^c^
Total Calls^d^ Range	4.16 (3.18) 0.11 0–13	3.75 (2.81) 0.10 0–15	3.83 (3.19) 0.11 0–15	F (2,2525) = 4.25; *p* = 0.014

^a^There was a statistically significant difference between the simultaneous and sequential group; *p* = 0.001

^b^Simultaneous vs. sequential

^c^Control vs. simultaneous

^d^Includes scheduled calls and participant initiated additional calls and healthy living calls

## Discussion

In this trial, we found that neither cessation nor weight change was improved by adding telephone weight management counseling to smoking cessation either sequentially or simultaneously, as compared to tobacco treatment alone. However, simultaneously adding weight management to cessation counseling reduced tobacco cessation in multiply imputed data. Others have suggested a potential undermining effect on tobacco abstinence when smoking cessation treatment simultaneously addresses both quitting smoking and preventing weight gain [30]. Findings of our current quitline effectiveness trial lend support to that cautionary note. It is plausible that key messages for assisting smokers with quitting tobacco were somewhat obscured by the added weight control intervention. Treatment fatigue could be another factor. The simultaneous group may have experienced a differential burden by having to concentrate on changing two difficult behaviors at the same time.

Our trial partially replicated findings from the prior efficacy trial conducted by Spring et al. (2004) in the respect that clinical outcomes were better for sequential than simultaneous treatment. In the Spring et al. (2004) study, however, the weight outcome showed an advantage for sequential treatment, whereas quit rates were comparable across all treatments. In the Spring et al. (2004) trial, reduced attendance and greater self-imposed treatment priorities for simultaneous treatment relative to cessation only and sequential treatments were the only evidence to suggest that simultaneous treatment imposed relatively greater burden than the other treatment conditions. In the present study, in contrast, poorer abstinence outcome in the simultaneous intervention condition showed them to be disadvantaged relative to the other groups. Also, whereas the Spring et al. (2004) study found a relative advantage favoring sequential treatment over simultaneous or control treatment for the weight outcome, no difference in weight change was observed among the treatment groups in the present study.

While the survey response rates at 6 months in the current study (43–49% across groups) were higher than response rates in the Spring et al. (2004) study at 9 months (37.5% across all groups), quit rates coding missing = smoking were comparable: 19–24.4% self-reported abstinence in the current study and 18–21% bioverified abstinence in the Spring et al. (2004) study. Both were higher than the 10–14% quit rate for the standard quitline [15, 31] and the 17.7% quit rates reported in our prior quitline studies [10, 32]. The observed 6 month (survey responder) quit rates in the current study (47–53.7%) were also higher than the observed quit rates from our two prior quitline studies (33.3% and 32–43%) [10, 32]. The number of standard quitline counseling sessions (calls 1–5) completed in the current study (2.56–2.83) were also higher than in prior quitline studies (1.66–2.21 calls); [10, 32]. Thus, we believe that overall, the study groups were not harmed by the interventions and in fact may have benefited from participation.

Our inability to fully replicate intervention effects described by Spring et al. (2004) could reflect difficulties in adequately implementing the sequential treatment condition, which disproportionately reduced the dose of weight management treatment given to that treatment group, perhaps preventing an adequate trial of the sequential treatment condition. The Spring et al. (2004) trial may have averted that implementation difficulty by providing a free 16 week tobacco cessation program and free meal replacement products to the simultaneous and sequential groups – functionally creating a delayed incentive that sequentially treated participants could not obtain unless they attended the delayed weight management treatment sessions.

The absence of a differential treatment effect on weight gain in the current study, as compared to the Spring et al. (2004) study may reflect floor effects arising from a lack of weight gain in any treatment group, including the controls. In fact, contrary to findings from prior smoking cessation trials, we found no significant differences in weight change among those who said they had quit and those who said they continued to smoke. Moreover, the mean change in self-reported weight gain among abstainers ranged from 0.35 ± 5.4 kg to 0.56 ± 5.9 kg across groups, which is less than the average directly measured weight gain observed in Spring’s trial (2.2–3.4 kg) and our prior two quitline studies using self-reported weight change: 3.1 ± 1.7 kg); [32] and 0.25 ± 8.3 kg (controls); − 0.5 ± 7.8 kg (weight concerns intervention) [10]. Importantly, rather than focusing on calorie regulation, the weight intervention used in the Bush et al. (2012) Weight Concerns study promoted healthy food choices and addressed excessive worry about gaining weight, replicating a prior weight concern intervention implemented by Perkins et al. [9].

The small weight gains reported by smokers in the present study could reflect measurement error or demand characteristics associated with the use of self-reported assessment of weight in a clinical trial that has weight as an outcome. Although plausible, several factors argue against that interpretation. First, there were no differences among treatment groups on the weight outcome, even though demand characteristics would be expected to bias the weight gain estimates downward more for the simultaneous and sequential weight treatments than for the cessation only treatment that did not address weight. Second, our results are consistent with a few recent studies which also reported relatively modest weight gain among smokers. For example, Scherr et al. (2015) found that 51% of those who quit for at least 24 months showed no significant change in weight (0.40 kg; SD ± 2.99 kg). The TARGIT study, a recent efficacy trial testing combined tobacco and weight management treatments also found no significant intervention effects or differences on tobacco abstinence or weight gain. Controls gained an average of 1.45 kg; the intervention group gained an average of 0.32 kg [33]. The weight gain reported in the Spring et al.. (2004) trial is more consistent with prior studies. Results from the Framingham Offspring Study used statistical modeling and showed that 13% of recent quitters gained more than 10 kg and that the average change in weight was 2.7 kg [34]. While Lycett et al. [5] found that post cessation weight gain averaged 8.79 kg over 8 years, results from a systematic review of 62 studies showed that the average weight gain among successful quitters was 4.67 kg at 12 months [35]. It is possible that demographic and intervention differences contributed to variation between the present results and those of the prior efficacy trial (Spring et al. 2004). Spring et al. 2004 enrolled more African Americans (31% vs our 25%) and 100% were female compared with 66% in the present study. The mean BMI in the prior study also was slightly lower (mean = 27) than in our sample (mean = 30). Although we delivered similar treatment components (tobacco cessation counseling and weight management) as in the Spring et al. (2004) study, we adapted them for a more resource-limited real-world context (e.g. by not providing free meal replacements, decreasing the number of treatment sessions, reducing the length of treatment sessions and making treatment individual- instead of group-based).

### Limitations and strengths

We did not exclude smokers who were of normal BMI since our prior quitline study found that baseline BMI was not a significant predictor of success in quitting tobacco [32]. Study participants were smokers seeking services from state or commercial quitlines and might not be representative of smokers who have not sought cessation treatment or those using other forms of cessation support. Given that this was an effectiveness trial within a real-world setting, the population was very similar to the quitline population. Like the general quitline population, study participants utilized only about half of the standard 5-call tobacco cessation calls offered, and call completion was a predictor of smoking cessation [31, 36, 37]. Low call completion continues to present challenges in treatment delivery. Another limitation of this study is our implementation problem which stemmed from the fact that the number of coaches trained to deliver tobacco is much larger than the number of coaches trained to deliver weight content. That, and the unforeseen influx of other business programs utilizing coaches trained on weight, caused limited availability of weight coaches which delayed delivery of the simultaneous Tobacco + Weight intervention calls (Calls 1–5) and the Sequential Weight only calls (Calls 6–10). On average it took longer between the initiation of Call 1 and completion of Call 5 for the simultaneous group, compared to the other two groups. Similarly, the delay in getting a call from a weight coach may have resulted in less exposure to the weight management treatment than expected for the sequential group because of the increased risk of drop out as the time between calls increased. Because of the lower response rate at 6 months for the simultaneous group and the sequential group receiving less weight management treatment than expected, the combined treatment groups no longer represented just the effect of weight management treatment (either sequential or simultaneous); it also represented the effect of implementation problems. Although there was a common cause (limited availability of coaches trained to deliver weight content), the effects were different on the two treatment groups due to the different scheduling of smoking and weight management calls. This raised significant issues with regard to interpreting a combined treatment effect. Consequently, we modified the analyses by allowing each treatment effect to be separately estimated.

Another potential limitation is that data on smoking and weight were self-reported without verification by direct objective measurement. Although biochemical validation of smoking is ideal, self-reported smoking is consistent with standard measures used for population-based interventions. Evidence suggests false reporting is minimal for low-intensity interventions with no face-to-face contact [38, 39]. Regarding the use of self-reported weight, the literature indicates that people tend to consistently underestimate their weight and their weight gain across time points, with underestimation disproportionately greater among the more overweight/obese [40]. Systematic under reporting of weight could explain the lower reported weight gains across groups in our study. To address these problems, we asked participants their current weight at baseline and follow up and used this data to calculate the weight gain using their self-reported weights. If participants consistently underestimate their weight by 10 pounds (4.5 kg), at baseline, 6 months and at 12 months, their calculated weight gain from baseline to 6 months or 12 months will still be correct (since the biases cancel). Regardless, it is possible that the bias could be heightened for the simultaneous and sequential groups compared with cessation only controls. “Use of self-reported weights could have contributed to the large standard deviations in weight outcomes (shown in Table 2) and this could potentially undermine the reliability of our results.” Although directly measured weights are ideal, we chose to collect self-reported weight by phone for three reasons. First, this mode of measurement is consistent with how users interact with a telephone quitline. Second, provision of wireless scales that could convey objective weight data would have been prohibitively expensive. Third, requiring participants to attend a clinic to obtain measured height, weight and smoking status would introduce respondent burden that would presumably decrease participation rates and make the enrolled sample less representative.

## Conclusions

This study addresses an important public health issue and provides new data suggesting that adding weight control to cessation treatment simultaneously may adversely impact quit rates. Importantly, all groups had better quit rates than what has been observed in the standard quitline. Whether administering weight control intervention sequentially, after smoking cessation, could optimize both tobacco abstinence and weight control cannot be inferred from the present study. The sequential treatment could not be successfully implemented because few participants remained engaged in treatment. This trial contributes to the science of tobacco treatment by describing quit rates and cessation related change in weight among male and female smokers seeking treatment through a telephone tobacco quitline, two thirds of whom were also offered weight management counseling. The trial demonstrates the importance as well as the difficulty of translating complex, costly behavior treatments into a real world setting.

## Abbreviations

BMI: Body Mass Index
Cpd: Cigarettes per day
Kg: Kilogram
MI: Multiple imputation; multiply imputed
NRT: Nicotine replacement therapy
OBS: Observed
SD: Standard deviation
SE: Standard error
